# The Utility of a Resting Electrocardiogram (ECG-PH Index) in Evaluating the Efficacy of Pulmonary Endarterectomy in Chronic Thromboembolic Pulmonary Hypertension

**DOI:** 10.3390/jcm12247621

**Published:** 2023-12-11

**Authors:** Michał Piłka, Szymon Darocha, Michał Florczyk, Rafał Mańczak, Marta Banaszkiewicz, Piotr Kędzierski, Dariusz Zieliński, Krzysztof Wróbel, Adam Torbicki, Marcin Kurzyna

**Affiliations:** 1 Chair and Department of Pulmonary Circulation, Thromboembolic Diseases and Cardiology, Center of Postgraduate Medical Education, European Health Center, ERN-LUNG Member, 05-400 Otwock, Poland; szymon.darocha@ecz-otwock.pl (S.D.); michal.florczyk@ecz-otwock.pl (M.F.); rafal.manczak@ecz-otwock.pl (R.M.); marta.banaszkiewicz@gmail.com (M.B.); piotr.kedzierski@ecz-otwock.pl (P.K.); adam.torbicki@ecz-otwock.pl (A.T.); marcin.kurzyna@ecz-otwock.pl (M.K.); 2 Cardiac Surgery Department, Medicover Hospital, 02-972 Warsaw, Poland; farok@wp.pl (D.Z.); krzysztof.wrobel17@gmail.com (K.W.); 3 Faculty of Medicine, Lazarski University, 02-662 Warsaw, Poland

**Keywords:** electrocardiography, pulmonary hypertension, chronic thromboembolic pulmonary hypertension, pulmonary endarterectomy

## Abstract

Background: The ECG-PH index (PH-ECG score) has been proposed as a valuable ECG-derived method of evaluating the effectiveness of balloon pulmonary angioplasty (BPA) in chronic thromboembolic pulmonary hypertension (CTEPH). Pulmonary endarterectomy (PEA) is the main form of therapy for CTEPH with a proximal clot location. The objective of this study was to assess the clinical utility of a resting electrocardiogram (ECG-PH index) in assessing the effectiveness of PEA in CTEPH patients. Methods: The retrospective analysis included 73 patients who underwent PEA. Their ECG-PH index values were calculated using four ECG parameters: R-wave amplitude V_1_ + S-wave amplitude V_5_/V_6_ > 10.5 mm, QRS-wave axis > 110 degrees, R-wave amplitude V_1_ > S-wave amplitude V_1_, and SIQIII pattern. PH-ECG scores were assessed after a median time of 13 months (IQR: 8–31 months) had passed since the PEA procedures. Results: The current analysis documented that ECG-PH index = 0 is a good reflection of mPAP < 25mmHg (sensitivity 76.1%; specificity 66.7%; positive predictive value 79.5%; negative predictive value 62.1%) or mPAP ≤ 20 mmHg (sensitivity 69.6%; specificity 70.6%; positive predictive value 88.6%; negative predictive value 41.4%) after PEA. The values of the area under the ROC curve for ECG-PH index were 0.772 (95% CI: 0.676–0.867) and 0.743 (95% CI: 0.637–0.849) for the mPAP < 25 mmHg and mPAP ≤ 20 mmHg patient groups, respectively. Conclusion: The ECG-PH index may be useful for monitoring the haemodynamic effect of PEA in CTEPH patients.

## 1. Introduction

Chronic thromboembolic pulmonary hypertension (CTEPH) is a form of pulmonary hypertension characterised by the presence of chronic thromboembolic material in the pulmonary vessels that persists after at least three months of effective therapy with antithrombotic drugs [1]. The cause of this disease entity is the fibrosis of embolic material remaining after an acute episode of pulmonary embolism, usually caused lower-extremity deep-vein thrombosis. Less commonly, organising thrombi in the pulmonary vessels may form on electrodes placed in the right heart cavities after the implantation of a pacemaker, defibrillator or cardiac resynchronisation system. It cannot be excluded that thrombi may also occasionally form in situ in the pulmonary arteries. The pathogenetic process involves disturbances in the coagulation system, endothelial cell dysfunction or platelet dysfunction, increasing blood thrombogenicity. Chronic narrowing of the pulmonary artery or arteries by thromboembolic lesions leads to an increase in pulmonary arterial pressure and the remodelling of the remaining pulmonary vessels, similar to that seen in pulmonary arterial hypertension. The consequence is a progressive increase in pulmonary vascular resistance, even when no further acute thromboembolic episodes occur. CTEPH is associated with progressive right ventricular failure, which has a significant negative impact on the patient’s quality and length of life.

Several therapies for CTEPH are currently available. One CTEPH therapy that can be used for patients experiencing thrombi in the pulmonary vessels with a proximal location is pulmonary endarterectomy (PEA). It is possible to perform PEA, depending on the experience of the institution, in about 50% of patients with CTEPH [2,3,4,5,6,7]. PEA involves the dissection of the pulmonary arterial wall and the removal of the internal membrane entrapped by organised thrombi that hinder blood flow [6,7]. According to the available data, in approximately 16–31% of patients who undergo PEA, CTEPH persists despite surgery [6,7]. Successful surgical treatment leads to relief of the right ventricle, whose normal function is an essential prognostic factor in patients with pulmonary hypertension. After treatment with PEA, patients require periodic non-invasive control and right heart catheterisation (RHC).

Based on the evolution of the electrocardiographic (ECG) curve, some studies have assessed the efficacy of intervention therapies and the prognostic value of selected ECG parameters of the right ventricular and right atrial hypertrophy and overload in CTEPH patients [8,9,10,11,12]. Among other aspects, the usefulness of the ECG-PH index (PH-ECG score) in evaluating the effectiveness of therapies in patients whose clots present in a distal location treated with balloon pulmonary angioplasty (BPA) has been investigated [12]. Currently, there are scarce data in the literature regarding the evaluation of the ECG curve for CTEPH patients who have undergone PEA [13]. The main purpose of this study was to assess the clinical utility of the ECG-PH index in evaluating the effectiveness of PEA in CTEPH patients with clots in a proximal location.

## 2. Materials and Methods

The study included CTEPH patients hospitalised at the European Health Centre in Otwock, Poland, between 2013 and 2023 who had undergone PEA. All patients were selected for treatment with PEA in accordance with the current standards [1,14,15,16]. Ultimately, the analysis included 73 patients who underwent functional (6 min walk test, laboratory tests), haemodynamic (right heart catheterisation) and electrocardiographic (resting ECG) assessment after a median time of 13 months (IQR: 8–31 months) has passed since the PEA procedure. We had no access to the results from patients’ assessment before PEA. Patients with arrhythmia and/or who had seen significant changes in their ECG curve secondary to coronary heart disease were excluded from the study. The Bioethics Committee approved the study protocol (decision number 88/PB/2015; 18 November 2015).

### 2.1. Right Heart Catheterisation and Pulmonary Endarterectomy

Right heart catheterisation was performed via internal jugular or femoral vein access in accordance with the applicable standards [17]. The following haemodynamic parameters were measured or calculated: mean right atrial pressure (RAP), systolic pulmonary arterial pressure (sPAP), mean pulmonary arterial pressure (mPAP), pulmonary vascular resistance (PVR), cardiac index (CI) and pulmonary capillary wedge pressure (PCWP).

PEA was performed by cardiac surgeons with the greatest experience in Poland in performing this kind of procedure (20–30 procedures per year).

### 2.2. 6 Min Walk and Laboratory Tests

A 6 min walk test (6MWT) was conducted by qualified medical personnel in accordance with the applicable standards of the American Thoracic Society [18]. Myocardial necrosis was assessed by determining the level of high-sensitivity troponin (Roche, Mannheim, Germany; plasma, normal values < 0.003 ng/mL), while the degree of cardiac failure was evaluated by determining the level of NT-proBNP (Roche, Mannheim, Germany; serum, normal values < 125 pg/mL).

### 2.3. Electrocardiogram

Each patient underwent a standard 12-lead ECG in a supine position, performed using a commercially available machine (Philips PageWriter TC50, Andover, MA, USA; paper speed was 25 mm/s; 1 mV = 10 mm). The ECG assessment was performed after a median time of 13 months (IQR: 8–31 months) had passed since the PEA procedure.

The electrocardiograms were analysed with respect to four ECG variables (R-wave V_1_ + S-wave V_5_/V_6_ > 10.5 mm, QRS-wave axis > 110 degrees, R-wave V_1_ > S-wave V_1_, SIQIII pattern), according to which the ECG-PH index (PH-ECG score) was validated, and whose clinical usefulness was confirmed in a group of CTEPH patients treated with BPA in our previous study [13]. Sensitivity, specificity, and positive and negative predictive values were determined for the ECG-PH index, which is the sum of four ECG parameters (R-wave amplitude V_1_ + S-wave amplitude V_5_/V_6_ > 10.5 mm, QRS-wave axis > 110 degrees, R-wave amplitude V_1_ > S-wave amplitude V_1_, SIQIII pattern) in the derivation cohort (Table 1).

The absence of all four variables in: resting ECG (ECG-PH index = 0) is a good reflection of patients with mPAP < 25 mmHg (sensitivity 100%; specificity 80%; positive predictive value 84%; negative predictive value 100%). The ECG-PH index was then confirmed in the validation cohort, which was represented by a separate, random pool of CTEPH patients who had also undergone BPA [12].

### 2.4. Statistical Analysis

Statistical analysis was performed with Stata, version 15.1 (StataCorp. 2017. Stata Statistical Software: Release 15. StataCorp LLC, College Station, TX, USA). Continuous variables with normal distribution were presented as means and standard deviations, while continuous variables with non-normal distribution were presented as medians and interquartile ranges. The *t*-test or Mann–Whitney test (depending on the distribution of the analysed variable as assessed with the Shapiro–Wilk test) was used to compare the continuous variables. The ECG-PH index was defined for all patients after the PEA procedure. Sensitivity and specificity, as well as positive and negative predictive values, were estimated. The receiver operating characteristic (ROC) curve was plotted for the ECG-PH index, which was defined as the sum of the four analysed ECG variables. The area under the ROC curve (AUC) was calculated with corresponding 95% confidence intervals (CI).

## 3. Results

### 3.1. Baseline Characteristics of the Study Cohort

The study included 73 CTEPH patients (mean age 54 ± 15.2) treated with PEA. The general characteristics of the study population, after cardiac surgery treatment with PEA, are shown in Table 2.

**Figure 1:**
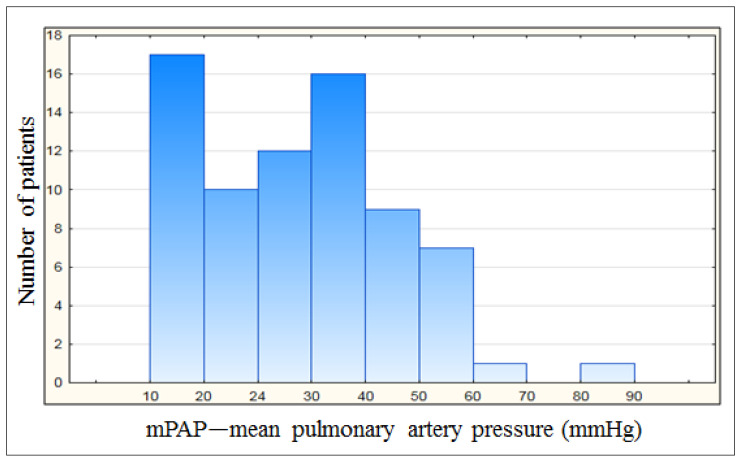
Number of patients relative to the achieved mPAP.

After PEA, 27 patients (median mPAP—20 mmHg /IQR 17–22/) achieved mPAP < 25 mmHg, including 17 patients who achieved mPAP ≤ 20 mmHg (median mPAP—18 mmHg /IQR 17–20/) (Figure 1).

### 3.2. Functional Parameters after PEA

Table 3 compares the functional parameters (NTproBNP, troponin, 6MWT) in groups of patients after PEA with mPAP ≥ 25 mmHg and <25 mmHg, mPAP > 20 mmHg and ≤20 mmHg, and mPAP < 25 mmHg and ≤20 mmHg.

### 3.3. ECG-PH Index Score after PEA Depending on mPAP

**Figure 2:**
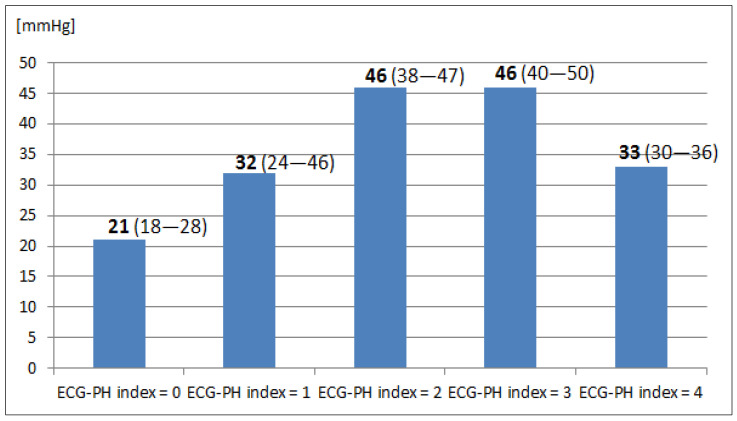
Median (IQR) values of mean pulmonary artery pressure (mPAP) for the ECG-PH index.

The median (IQR) values of mPAP for ECG-PH index 0, 1, 2, 3 and 4 were 21 mmHg (IQR 18–28), 32 mmHg (IQR 24–46), 46 mmHg (IQR 38–47), 46 mmHg (IQR 40–50) and 33 mmHg (IQR 30–36), respectively (Figure 2).

The absence of all four ECG variables (R-wave V_1_ + S-wave V_5_/V_6_ > 10.5 mm, QRS-wave axis > 110 degrees, R-wave V_1_ > S-wave V_1_, SIQIII pattern) in resting ECG (ECG-PH index = 0) was observed in patients who had mPAP < 25 mmHg (sensitivity 76.1%; specificity 66.7%; positive predictive value 79.5%; negative predictive value 62.1%) or mPAP ≤ 20 mmHg (sensitivity 69.6%; specificity 70.6%; positive predictive value 88.6%; negative predictive value 41.4%) after cardiac surgery treatment with PEA (Table 4 and Table 5).

**Figure 3:**
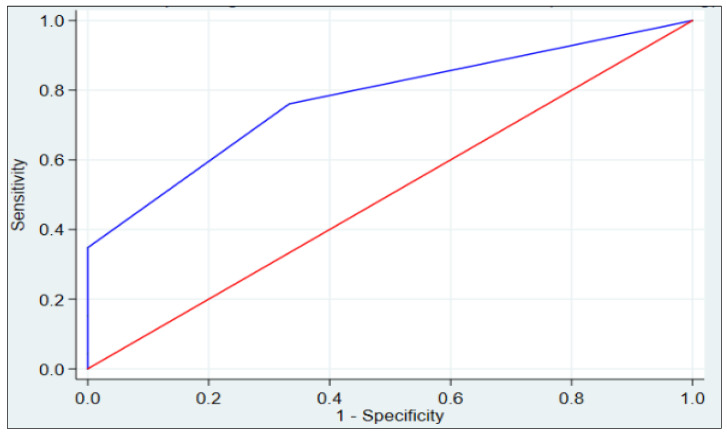
ROC curve for the ECG-PH index in the group of patients with mean pulmonary artery pressure (mPAP) < 25 mmHg.

The area under the ROC curve for the ECG-PH index in the group of patients with mPAP < 25 mmHg was 0.772 (95% CI: 0.676–0.867) (Figure 3).

**Figure 4:**
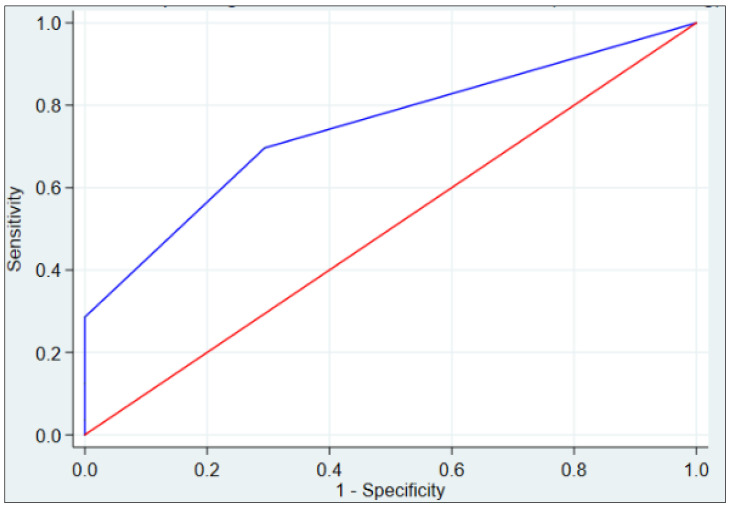
ROC curve for the ECG-PH index in the group of patients with mean pulmonary artery pressure (mPAP) ≤ 20 mmHg.

The area under the ROC curve for the ECG-PH index in the group of patients with mPAP ≤ 20 mmHg was 0.743 (95% CI: 0.637–0.849) (Figure 4).

## 4. Discussion

PEA is a treatment modality for CTEPH patients with a proximal thrombi localisation that results in good long-term survival. Haemodynamically successful PEA often results in the normalization of mPAP. However, even if the normalization of haemodynamics is not achieved, the clinical gain for the patient is still significant [19].

This study is the first to evaluate the clinical utility of a simple ECG-PH index (PH-ECG score) in assessing the long-term haemodynamic effect of surgical treatment of CTEPH patients with a proximal clot location.

The diagnostic value of resting ECG in pulmonary hypertension is well documented [20,21,22].

Pre-cardiac leads, especially V_1_, V_5_ and V_6_, are very valuable in the electrocardiographic diagnosis of pulmonary hypertension. The following haemodynamic parameters have demonstrated a high diagnostic value (predictive value of more than 80%) for pulmonary hypertension: an R/S in V_1_ of more than one, an R/S V_6_ of less than one, a QRS axis of more than 110 degrees, a qR in V_1_. Particularly important in the prediction of high pulmonary hypertension is QRS axis more than 110 degrees (PASP ≥ 60 mmHg) [21]. The diagnostic value of the above electrocardiographic parameters was also confirmed in our work when validating the ECG-PH index (R-wave V_1_ + S-wave V_5_/V_6_ > 10.5 mm, QRS-wave axis > 110 degrees, R-wave V_1_ > S-wave V_1_, SIQIII pattern). However, it should be borne in mind that a normal resting electrocardiogram cannot entirely exclude pulmonary hypertension. For this reason, there have been attempts to use additional electrocardiographic variables that would improve the sensitivity of this method. Additional information for patients with normal resting electrocardiogram and who have significant clinical probability of pulmonary hypertension can be provided by right-sided chest ECG (V3R-V5R). R-wave amplitude (AUC 0.802; *p* < 0.001) and R/S ratio (AUC 0.823; *p* < 0.001) in V5R were found to be good predictors of pulmonary hypertension [22].

Several studies have also demonstrated that effective treatment targeted at pulmonary arterioles in PAH patients resulted in significant changes in the ECG trace [23,24,25].

The diagnostic value of the *p*-wave amplitude in lead II and the T-wave axis was confirmed. ROC analysis confirmed that the *p* amplitude in lead II (AUC 0.8; *p* = 0.01), the QRS axis (AUC 0.7; *p* = 0.03), and the T axis (AUC 0.9; *p* < 0.001) were significant determinants of treatment response [24]. The resting electrocardiogram not only plays a role in the diagnosis and assessment of the effects of pulmonary hypertension therapy, but its usefulness has also been confirmed in terms of the prognosis of patients with pulmonary hypertension. The presence of qR syndrome in V_1_ has been shown to be related to a significantly advanced form of pulmonary hypertension with associated right ventricular failure and increased risk of death [25].

The diagnostic value of the resting ECG was also evaluated in patients with acute pulmonary embolism, not only with regard to the prognosis in the acute phase but also to screening for CTEPH [26,27,28,29,30,31,32]. In addition, the ECG curve was also examined for its ability to differentiate between proximal and distal CTEPH localization [33].

The presence of negative T waves in leads V_1_–V_3_ has been shown to be associated with right ventricular dysfunction in patients with acute pulmonary embolism, with subsequent consequences in the poorer prognosis of this group of patients. Similarly, this relates to the presence of S1Q3T3 and right bundle branch block, with good specificity but only moderate accuracy [26]. The presence of electrocardiographic signs of right ventricular overload during acute pulmonary embolism is associated with higher diagnosis of CTEPH in this group of patients [27].

Recent studies on acute pulmonary thromboembolism have assessed and compared various ECG scores for use in patients undergoing diagnostics due to suspected acute pulmonary embolism, e.g., Daniel-ECG score and novel ECG score [29,30]. However, both scales require the analysis of a large number of electrocardiographic parameters, making them difficult to use in everyday clinical practice. Compared to the above scales, ours is a simple tool for monitoring CTEPH patients undergoing PEA.

The use of intervention treatment with BPA in CTEPH patients is associated with significant changes in ECG results [8,9,10,11,12].

An interesting study by Ayhan Kol et al. confirmed the usefulness of ECG in monitoring haemodynamic improvement in CTEPH patients with a distal clot location treated with BPA: specifically, the value of changes in the T-wave amplitude in the V_2_ lead [9]. Nishiyama et al. observed the evolution of the ECG curves of CTEPH patients during treatment with BPA [8]. The study confirmed that the value of the S-wave amplitude in lead V_5_, the sum of the R-wave amplitude in lead V_1_ and the S-wave amplitude in lead V_5_, the S-wave amplitude in lead I, and the QRS axis were important predictors of mean pulmonary arterial pressure ≥ 30 mmHg. Similar conclusions were reached by Yokokawa et al., who applied 15 ECG criteria for right ventricular hypertrophy according to the recommendations of the American Heart Association. The mean number regarding the right ventricular hypertrophic criteria was significantly reduced after BPA procedures (4.8 ± 2.6 to 3.1 ± 2.5; *p* = 0.003). The number of patients who met the criteria of deep S in V_6_ (*p* = 0.005) and max R in V_1,2_ + max S in I, aV_L_-S in V_1_ (*p* = 0.046) decreased significantly after balloon pulmonary angioplasty [10].

Our two previous studies analysing the ECG curve confirmed not only the trace variability of haemodynamically effective BPA therapy but also its usefulness in monitoring the therapy and its long-term effects [11,12]. One of these studies revealed that only haemodynamically effective BPA has a significant effect regarding changes in the ECG curve. In the analysis of ECG variables, statistical significance was demonstrated for the following parameters: T-wave axis (*p* < 0.001), *p* wave in lead II (*p* < 0.001), S wave in lead V_5_ (*p* < 0.001) and R/S ratio in lead V_5_ (*p* < 0.001) [11]. The other study, based on four ECG variables (R-wave amplitude V_1_ + S-wave amplitude V_5_/V_6_ > 10.5 mm, QRS-wave axis > 110 degrees, R-wave amplitude V_1_ > S-wave amplitude V_1_, SIQIII pattern), validated and then confirmed the value of the ECG-PH index (PH-ECG score) in monitoring patients with CTEPH and a distal clot location treated with repeated BPA procedures. The absence of all four variables in resting ECG (ECG-PH index = 0) is a good reflection of patients with mPAP < 25 mmHg (sensitivity 100%; specificity 80%; positive predictive value 84%; negative predictive value 100%) [12].

Currently, very interesting attempts are being undertaken to predict precapillary pulmonary hypertension based on selected electrocardiographic parameters. In a retrospective study by Tokgöz et al. of 562 patients who had undergone right heart catheterisation, an attempt was made to determine which electrocardiographic variables could predict precapillary pulmonary hypertension. *p*-wave amplitude, R in aVR, right axis deviation or indetermination and R/Sr in V_1_ and V_2_ correlated significantly with pulmonary haemodynamics. *p*-wave amplitude, R-wave in aVR, QRS axis and R/Sr in V_1_ may all play a role in predicting precapillary pulmonary hypertension [34].

Analysis of the electrocardiographic curve during exercise is very interesting. Significant changes in the ST-T segment during exercise in patients with pulmonary hypertension have been shown to be associated with a higher likelihood of death or lung transplantation. Changes in the ST-T segment during exercise correlate significantly with high pulmonary arterial pressure (mPAP > 55 mm) [35].

Considering the scarcity of data on the analysis of the ECG curve in CTEPH patients with a proximal clot location who have undergone cardiac surgery with PEA, the above analysis verifies the use of the ECG-PH index in this group of patients. The only existing publication demonstrating the variability of the ECG curve after treatment with PEA was a study by Ghio et al. [13]. The study, however, focused on the fact that ECG indices better reflect the haemodynamic overload (*p*-wave amplitude in DII, R-wave amplitude in V_1_, number of patients with negative T wave in V_1_–V_3_) and pathological remodelling of the right ventricle (S-wave amplitude in V_1_, R:S wave ratio in lead V_6_, prevalence of SIQIII pattern).

Our study also confirmed the value of the electrocardiographic parameters in the range of the pre-cardiac leads V_1_, V_5_ and V_6_ and the prevalence of the SIQIII pattern. However, the *p*-wave amplitude was not included in the set of electrocardiographic parameters, on the basis of which the ECG-PH index was defined.

In summary, our paper is unique since it shows how a simple ECG index (i.e., the ECG-PH index), validated and verified on a population of CTEPH patients treated with BPA, may be helpful in monitoring CTEPH patients with a proximal clot location who are treated with PEA.

### Study Limitations

The first limitation of this study was the lack of insight into the functional and haemodynamic assessment of patients before the PEA procedures. Additionally, only a small group of patients were included in the analysis. Another limitation was the lack of assessment of the right ventricular morphology and function with a precise imaging technique, such as MRI or 3D ECHO.

## 5. Conclusions

A simple ECG scale (ECG-PH index) may be useful for monitoring the long-term haemodynamic effect of surgical treatment (PEA) for CTEPH patients.

## Figures and Tables

**Table 1:** ECG-PH index—the sum of the occurrence of four parameters.

ECG-PH Index	R-Wave V_1_ + S-Wave V_5_/V_6_ > 10.5 mm	QRS-Wave Axis > 110 Degrees	R-Wave V_1_ > S-Wave V_1_	SIQIII Pattern
ECG-PH index = 0 (0 + 0 + 0 + 0)	0	0	0	0
ECG-PH index = 1 (1 + 0 + 0 + 0)	presence of any one parameter			
ECG-PH index = 2 (1 + 1 + 0 + 0)	presence of any two parameters			
ECG-PH index = 3 (1 + 1 + 1 + 0)	presence of any three parameters			
ECG-PH index = 4 (1 + 1 + 1 + 1)	1	1	1	1

**Table 2:** General characteristics of study cohort after pulmonary endarterectomy (PEA).

General Characteristics of the Population	Patients after PEA (*n* = 73)
Female sex, *n* (%)	26 (35.6)
Age, mean (SD)	54 (15.2)
WHO functional class, *n* (%)	
I	10 (13.7)
II	31 (42.5)
III	28 (38.4)
IV	4 (5.5)
NTproBNP (pg/mL), median (IQR)	279 (148.5–757.9)
Troponin (ng/mL), median (IQR)	0.008 (0.006–0.014)
6MWT (m), mean (SD)	412 (161.4)
mPAP (mmHg), median (IQR)	29 (21–40)
sPAP (mmHg), median (IQR)	47 (35–66)
CI (L/min/m^2^), median (IQR)	2.56 (2.17–2.98)
PVR (Wood units), median (IQR)	3.95 (2.15–6.76)
PCWP (mmHg), median (IQR)	10 (8–12)
mRAP (mmHg), median (IQR)	6 (4–10)
Sildenafil-targeted therapy n (%)	7 (9.6)
Riociguat-targeted therapy n (%)	9 (12.3)

**Table 3:** Comparison of functional parameters (NTproBNP, troponin, 6MWT) in three groups of patients after PEA.

Functional Parameters	mPAP		*p*-Value	mPAP		*p*-Value	mPAP		*p*-Value
	≥25 mmHg	<25 mmHg		>20 mmHg	≤20 mmHg		<25 mmHg	≤20 mmHg	
pNTproBNP (pg/mL) median (IQR)	570.2 (187.2–1297)	167.4 (106.4–266.3)	***p* < 0.001**	386 (172.6–1218)	158.7 (106.4–255)	***p* = 0.003**	167.4 (106.4–266.3)	158.7 (106.4–255)	*p* = 0.96
Troponina (ng/mL) median (IQR)	0.01 (0.006–0.15)	0.006 (0.005–0.013)	*p* = 0.057	0.008 (0.006–0.0145)	0.007 (0.005–0.012)	*p* = 0.23	0.006 (0.005–0.013)	0.007 (0.005–0.012)	*p* = 0.95
6MWT (m) mean (SD)	372 (145.9)	490 (165)	***p* = 0.004**	401.4 (153.2)	455.3 (193)	*p* = 0.28	490 (165)	455.3 (193)	*p* = 0.7

**Table 4:** Sensitivity, specificity, positive predictive value (PPV) and negative predictive value (NPC) for the ECG-PH index in patients with mPAP < 25 mmHg.

ECG-PH Index	mPAP < 25 mmHg	mPAP ≥ 25 mmHg	Sensitivity	Specificity	PPV	NPV	Youden Index
ECG-PH index = 0	18	11	76.1	66.7	79.5	62.1	42.8
ECG-PH index = 1	9	19	34.8	100.0	100.0	47.4	34.8
ECG-PH index = 2	0	9	15.2	100.0	100.0	40.9	15.2
ECG-PH index = 3	0	5	4.3	100.0	100.0	38.0	4.3
ECG-PH index = 4	0	2	0.0	100.0	100.0	0.0	0.0

**Table 5:** Sensitivity, specificity, positive predictive value and negative predictive value for the ECG-PH index in patients with mPAP ≤ 20 mmHg.

ECG-PH Index	mPAP ≤ 20 mmHg	mPAP > 20mmHg	Sensitivity	Specificity	PPV	NPV	Youden Index
ECG-PH index = 0	12	17	69.6	70.6	88.6	41.4	40.2
ECG-PH index = 1	5	23	28.6	100.0	100.0	29.8	28.6
ECG-PH index = 2	0	9	12.5	100.0	100.0	25.8	12.5
ECG-PH index = 3	0	5	3.6	100.0	100.0	23.9	3.6
ECG-PH index = 4	0	2	0.0	100.0	100.0	0.0	0.0

## Data Availability

Data available from the authors upon request.
